# Reproductive gene expression in a coral reef fish exposed to increasing temperature across generations

**DOI:** 10.1093/conphys/cox077

**Published:** 2018-01-06

**Authors:** Heather D Veilleux, Jennifer M Donelson, Philip L Munday

**Affiliations:** 1 ARC Centre of Excellence for Coral Reef Studies, James Cook University, Townsville QLD 4811, Australia; 2 School of Life Sciences, University of Technology Sydney, PO Box 123, Broadway, NSW 2007, Australia

**Keywords:** *Acanthochromis polyacanthus*, climate change, gonadotropins, qRT-PCR, reproduction, transgenerational plasticity

## Abstract

Reproduction in marine fish is generally tightly linked with water temperature. Consequently, when adults are exposed to projected future ocean temperatures, reproductive output of many species declines precipitously. Recent research has shown that in the common reef fish, *Acanthochromis polyacanthus*, step-wise exposure to higher temperatures over two generations (parents: +1.5°C, offspring: +3.0°C) can improve reproductive output in the F2 generation compared to F2 fish that have experienced the same high temperatures over two generations (F1 parents: +3.0°C, F2 offspring: +3.0°C). To investigate how a step-wise increase in temperature between generations improved reproductive capacity, we tested the expression of well-known teleost reproductive genes in the brain and gonads of F2 fish using quantitative reverse transcription PCR and compared it among control (+0.0°C for two generations), developmental (+3.0°C in second generation only), step (+1.5°C in first generation and +3.0°C in second generation), and transgenerational (+3.0°C for two generations) treatments. We found that levels of gonadotropin receptor gene expression (*Fshr* and *Lhcgr*) in the testes were reduced in developmental and transgenerational temperature treatments, but were similar to control levels in the step treatment. This suggests *Fshr* and *Lhcgr* may be involved in regulating male reproductive capacity in *A. polyacanthus*. In addition, lower *Fshb* expression in the brain of females in all temperature treatments compared to control, suggests that *Fshb* expression, which is involved in vitellogenesis, is sensitive to high temperatures. Our results help elucidate key genes that facilitate successful reproduction in reef fishes when they experience a gradual increase in temperature across generations consistent with the trajectory of climate change.

## Teaser

We explored the potential underlying mechanisms of improved reproduction in a coral reef fish when exposed to a step-wise increase in temperature across generations. Expression of reproductive genes revealed that gonadotropin receptor genes, *Lhcgr and Fshr*, in male gonads are associated with reproductive plasticity.

## Introduction

Climate change is predicted to raise tropical sea surface temperatures by as much as 3°C by 2100 (Collins *et al.*, 2013) with profound implications for the function and productivity of marine ecosystems (Harley *et al.*, 2006; Pörtner *et al.*, 2014). While many species will shift their geographic ranges as the oceans warm (Poloczanska *et al.*, 2013), the populations that remain within the current range will experience elevated temperatures in the future. Adaptation to warmer conditions could occur if a population has enough standing genetic variation (Munday *et al.*, 2013), although there is concern that for many species the time required for genetic evolution may exceed the rate of ocean warming (Parmesan, 2006). Acclimation through phenotypic plasticity could be another important process that will assist organisms in coping with climate change (Huey *et al.*, 2012; Munday *et al.*, 2013; Crozier and Hutchings, 2014; Merila and Hendry, 2014). Beneficial acclimation occurs when physiological, morphological or behavioural phenotypes are plastically altered to better suit the environment (Angilletta, 2009). The phenotype of many animals can be adjusted in response to short-term changes in environmental conditions, such as daily or seasonal environmental fluctuations (reversible acclimation; Angilletta, 2009). However, environmental conditions experienced during early ontogeny can also induce phenotypic changes that persist throughout life (developmental plasticity) and parental exposure can alter the performance of their offspring in the same environment (transgenerational plasticity; Salinas *et al.*, 2013; Torda *et al.*, 2017).

Reproduction in fishes is tightly regulated by temperature, influencing processes such as gametogenesis, ovulation and spermiation, embryogenesis and hatching, larval development, and sex determination (Van der Kraak and Pankhurst, 1997; Pankhurst and Munday, 2011). As fish are ectothermic and lack internal thermal regulation (Fry, 1967), changes in environmental temperature can have serious impacts on these critical reproductive processes (Davies *et al.*, 1986; Van der Kraak and Pankhurst, 1997; Pankhurst and Munday, 2011; Zeh *et al.*, 2012). Specifically, changes in environmental temperature are known to influence reproductive processes in numerous species of fish via the hypothalamo-pituitary-gonadal (HPG) axis. Following a temperature cue, gonadotropin-releasing hormones (GnRH) are synthesized in the hypothalamus and synaptically released onto gonadotropic cells in the pituitary, stimulating the release of pituitary gonadotropins: follicle stimulating hormone (FSH) and lutenising hormone (LH) (reviewed by Planas and Swanson, 2008; Levavi-Sivan *et al.*, 2010; Zohar *et al.*, 2010). In many fishes, dopamine (DA) has been shown to play an inhibitory role in releasing the gonadotropins, suggesting that FSH and LH release is dependent on the balance between DA and GnRH (reviewed by Dufour *et al.*, 2010). FSH and LH stimulate gonadal function in both males and females by regulating the production of sex steroids (steroidogenesis) and gamete maturation (spermatogenesis and oogenesis, respectively). In male gonads, the enzyme Cyp11b1 converts the less active testosterone into 11-ketotesterone (11-KT). In ovaries, the enzyme Cyp19a1a (aromatase) converts testosterone to 17β-estradiol (E_2_). Plasma levels of LH and FSH, in addition to the presence of their receptors in the gonads, vary depending on the sex, the level of sexual maturation of the fish, the phase of spermatogenesis or oogenesis, and the species (Planas and Swanson, 2008; Levavi-Sivan *et al.*, 2010).

Due to the energetic cost and benefits of physiological optimization associated with reproduction, many species have evolved to reproduce within a narrow thermal range (Van der Kraak and Pankhurst, 1997; Browne and Wanigasekera, 2000; Visser *et al.*, 2009). Although some species have already shifted their reproductive timing due to current changes in temperature (Parmesan and Yohe, 2003), other species may not have this ability and declines in quality and/or quantity of offspring, or reduced capacity for reproduction in general, are observed at temperatures outside the optimal thermal range (Giebelhausen and Lampert, 2001; Donelson *et al.*, 2010; Miller *et al.*, 2015). Consequently, warming associated with climate change poses a significant risk to population sustainability in these species. At the molecular level, higher than optimal reproductive temperatures can suppress expression of reproductive hormones and steroids (e.g. King *et al.*, 2003; Pankhurst and King, 2010; Pankhurst and Munday, 2011). For example, when red seabream *Pagrus major*, were exposed to elevated temperatures for up to 10 days, brain mRNA levels of *GnRH1*, and pituitary mRNA levels of *GnRH-R*, *FshB* and *LhB* were reduced and there were lower serum levels of E_2_ (Okuzawa and Gen, 2013). Similarly, when reproductively active adult pejerrey (*Odontesthes bonariensis*) were exposed to elevated temperatures for 8 days, there were declines in transcript levels of *Gnrh1* (brain) and *FshB* (pituitary) in both sexes, *LhB* (pituitary) in males, and *Fshr, Lhr* and *Cyp19a1a* in female gonads, and reductions in plasma sex steroids (E_2_ and testosterone in females, 11-KT in males; Elisio *et al.*, 2012). However, the effects of longer-term (i.e. developmental or transgenerational) exposure to high temperatures on transcript abundance of reproductive genes in the brain or gonads in these or other species have yet to be evaluated.

Recent studies have investigated the plasticity of physiological traits in marine fishes following developmental or transgenerational exposure to projected future warming (Donelson *et al.*, 2011, 2012, 2016; Salinas and Munch, 2012; Shama and Wegner, 2014; Veilleux *et al.*, 2015). However, few have assessed the potential for reproductive plasticity when exposed to elevated temperatures. Recently, Donelson *et al.* (2016) demonstrated that the coral reef damselfish, *Acanthochromis polyacanthus*, has the capacity for transgenerational reproductive plasticity when exposed to higher temperatures in a step-wise fashion over two generations, +1.5°C in the first generation and then +3.0°C in the second generation. In contrast, fish that were exposed to +3.0°C for two generations ceased to reproduce at all. Our study aimed to evaluate differences in gene expression between adult *A. polyacanthus* that possessed differences in reproductive capacity due to developmental and transgenerational exposure to elevated temperature. Importantly, we assessed gene expression of fish in the same step-wise transgenerational temperature treatment that was shown by Donelson *et al.* (2016) to possess partial acclimation of reproductive capacity. We predicted that the expression of reproductive genes in the brains and gonads would be downregulated in fish that were exposed to the same high temperature as their parents (i.e. two generations at +3.0°C) as no fish in this treatment were able to reproduce. By contrast, we predicted that expression of reproductive genes in the step-wise treatment would be more similar to that of the controls, because they exhibited partial reproductive acclimation.

## Material and methods

### Study species and experimental design

Eight breeding pairs of *A. polyacanthus* (F0) were collected from the Palm Island region of the Great Barrier Reef, Australia, in July 2007. The Palm Island reefs are in the middle of the species range (18°37′S, 146°30′E) and have average yearly temperatures from 23.2°C to 28.5°C (Australian Institute of Marine Science temperature loggers 6–8 m; http://data.aims.gov.au). Breeding pairs were maintained in 60 L aquaria inside an environmentally controlled facility at James Cook University, Townsville, Australia.

The wild pairs produced offspring (F1) from December 2007 to February 2008. At 30 days post-hatching, clutches of F1 fish from each breeding pair were equally divided into one of three seasonally cycling temperature treatments: +0.0°C as well as +1.5°C and +3.0°C above average current seasonal temperatures (see Donelson *et al.* 2011 for more details). For 1 year after hatching, sibling fish were kept in groups of six in 40 L aquaria and then were reduced into pairs by the experimenter to reduce tank density. Mortality was very low among siblings, with >90% survival in all treatments. At 1.5 years post-hatching, fish were rearranged into non-sibling pairs from individuals from the same treatment, using an even number of individuals from each parental line. Fish reached maturity at 2 years old and reproduced during the Austral summer 2009–2010, generating 7, 7 and 3 clutches in the +0.0, +1.5 and +3.0°C F1 treatments, respectively.

**Figure 1:**
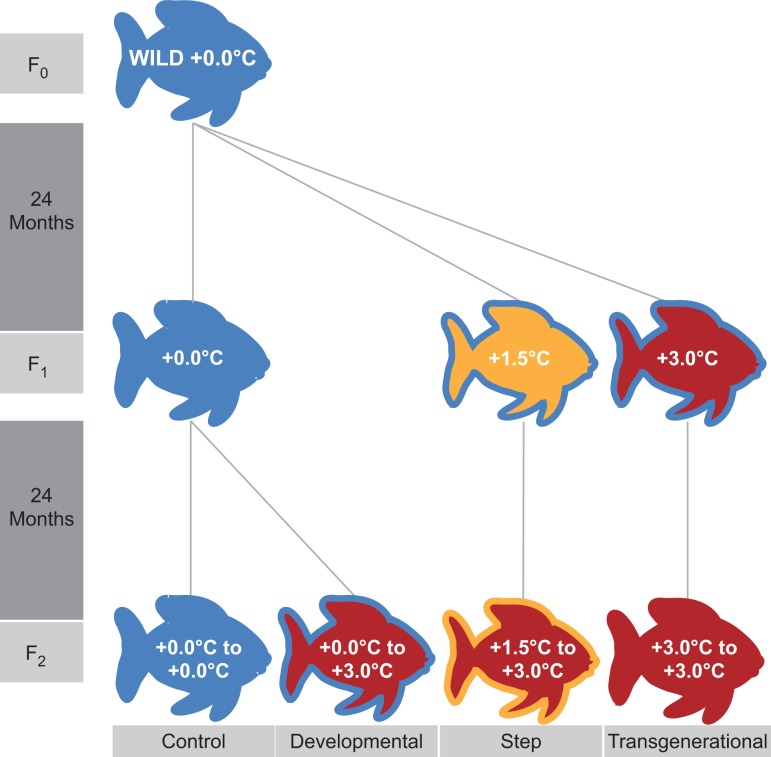
Experimental design tree showing three generations (F_0_, F_1_ and F_2_) of *Acanthochromis polyacanthus*. Temperature treatments are colour-coded with filled colours representing temperatures experienced for that generation and borders representing temperatures their parents experienced. Experimental duration for each generation is shown in the vertical dark grey bars to the left and treatments are indicated in the horizontal light grey bars below.

Thirty F2 juveniles from each clutch, were transferred to 30 L tanks immediately following hatching. Four F2 treatments were produced: (1) control +0.0°C, (2) developmental +3.0°C, (3) step +3.0°C and (4) transgenerational +3.0°C. Control and developmental treatments had parents reared at +0.0˚C, the step +3.0°C treatment had parents reared at +1.5°C, and the transgenerational fish had parents reared at +3.0°C. The full experimental design is shown in Fig. 1. At ~6 months of age, each sibling group was divided among four 40 L tanks (i.e. groups of 6–8 individuals per tank) to accommodate increased body size throughout development. At ~1.5 years old, all F2 fish were rearranged into non-sibling breeding pairs per 40 L tank (10, 9, 9 and 13 pairs for control, developmental, step and transgenerational, respectively) and kept in their respective temperatures (control = +0.0°C; developmental, step, transgenerational = +3.0°C). During the austral summer 2011–2012, nesting sites in each tank were checked daily for the presence of eggs.

Fish in all generations were maintained at the natural seasonal light-dark cycles for the collection location and were fed *ad libitum* twice per day on aquaculture feed of appropriate size for their development (Primo Aquaculture NRD 3/4, 5/8, and G12 pellets).

### mRNA quantification

In April 2012 at ~2 years old, a random subset of 4–5 adult individuals per F2 treatment were sacrificed and whole brains and gonads were immediately dissected, snap-frozen in liquid nitrogen, and stored at −80°C until processing. Tank temperatures remained at the summer breeding temperature. Whole brains and gonads were homogenized and applied to PerfectPure Preclear columns (VWR, Murarrie, Australia). RNA was extracted according to manufacturer instructions, including an on-column DNAse treatment. Total RNA quality and quantity was determined by absorbance readings on a NanoDrop Spectrophotometer (Invitrogen, Mulgrave, Australia) and an RNase-free 1% agarose gel.

Total RNA for brains and gonads was normalized to a common concentration of 200 and 40 ng μl^−1^, respectively. Complementary DNA (cDNA) was synthesized using 1 and 0.6 μg total brain and gonad RNA, respectively, and a blend of oligo(dT) and random primers in the iScript Reverse Transcription Supermix (Bio-Rad Laboratories, Gladesville, Australia), as per manufacturer's instruction. Each cDNA sample was 5-fold serially diluted twice in molecular grade water (Invitrogen, Mulgrave, Australia) to use as a working stock for quantitative reverse transcription PCR (qRT-PCR). Aliquots of each original brain cDNA sample were combined and five 1:5 dilutions were performed to generate samples for a standard curve and for calculating PCR efficiency for each brain primer pair. This procedure was repeated separately for the gonad cDNA samples.

Intron-spanning primers for five reproductive genes in the brain (*Fshb*, *Lhb*, *Gnrh1*, *Gnrhr* and *Ddc*), and four reproductive genes in the gonads (*Fshr*, *Lhcgr*, *Cyp19a1a* and *Cyp11b1*) were designed using 3Prime (Koressaar and Remm, 2007; Untergasser *et al.*, 2012) based on the genes from the assembled genome for *A. polyacanthus* (Schunter *et al.*, 2016). In addition, intron-spanning reference gene primers were designed based on the most stably expressed genes in the *A. polyacanthus* transcriptome (see supplementary information Veilleux *et al.*, 2015; Dvl1, Sin3b, Cnot1). Prior to the availability of the genome or transcriptome, primers for two reference genes (*Ef1a* and *18s rRNA*) were designed using 3Prime (Koressaar and Remm, 2007; Untergasser *et al.*, 2012) and were based on conserved regions of teleost genes obtained from the GenBank Public Database (Altschul *et al.*, 1997). Primer sequences and details are listed in Table 1.

**Table 1:** Quantitative reverse transcription PCR (qRT-PCR) brain and gonad target and reference genes, associated forward and reverse primer sequences and expected product length

Type	Gene		Forward Primer (5′−3′)	Reverse Primer (5′−3′)	Expected product (bp)
Brain	*Ddc*	Dopa Decarboxylase (an enzyme in the pathway that produces dopamine)	GTCCAGGCAACCAACTCCAG	CCTCCAATCAGAGCAGCTCG	110
	*Fshb*	Follicle Stimulating Hormone, Beta Polypeptide	CACCACCGTGTGTTCAGGAC	ACCTCGTAGGACCAGTCACC	105
	*Gnrh1/Sb-Gnrh*	Gonadotropin-Releasing Hormone 1	CTGTCAGCACTGGTCGTATG	ACTGAAGGGTGCGTCCAT	115
	*Gnrhr*	Gonadotropin-Releasing Hormone Receptor	TTCCTGCTCCCACTGGTCAT	GAGTCTTCATCCGGGCTCTG	148
	*Lhb*	Luteinizing Hormone, Beta Polypeptide	AGACGGTGTCTCTGGAGAAG	TACAGGTCCTGGTAGGTGC	148
Gonad	*Cyp11b1*	Cytochrome P450, Family 11, Subfamily B, Polypeptide 1 (a.k.a. 11b-hydroxylase)	CAGCACAGCAAGGGAGTCTT	CAGAAATCCCTCGCCACCTC	137
	*Cyp19a1a*	Cytochrome P450, Family 19, Subfamily A, Polypeptide 1 (a.k.a arom/aromatase)	CCGGACAGAGTTCTTCCTCA	CGAATGGCTGGAAGTAACGG	86
	*Fshr*	Follicle Stimulating Hormone Receptor	CCTCTCATCACCGTCTCCGA	CGGGTGAAGAAGGCGTACAG	95
	*Lhcgr*	Luteinizing Hormone/Choriogonadotropin Receptor	TGAACCTGGCTAGAAACGGC	AGAACTCGGACCTGTGGCTC	143
Reference	*Cnot1*	CCR4-NOT Transcription Complex, Subunit 1	ATCCACAACAAGGGCAGCACCC	TCAGTGTCCAGGTCCACAGCCA	95
	*Dvl1*	Dishevelled Segment Polarity Protein 1	AGTGAATCCGAGCCAGGTGTCC	ACTGTGACTGGAGACGGCATGG	93
	*Sin3b*	SIN3 Transcription Regulator Family Member B	AACAGGGACGCAACGGCTCT	TGGATGGTGGGCTGACGCTT	133
	*18s rRNA*	18s ribosomal RNA	TTGACGGAAGGGCACCACCA	AGAACGGCCATGCACCACCA	142
	*Ef1a*	Eukaryotic Translation Elongation Factor 1 Alpha 1	ACGCCTGGGTGCTGGACAAA	GCGACAATCAGCACAGCGCA	183

qRT-PCR was performed in triplicate 15 μl reactions using 1x SsoFast EvaGreen Supermix (Bio-Rad Laboratories, Gladesville, Australia), 0.3 μM forward and reverse primers, and 10 ng cDNA. Using the Rotor-Gene Q (Qiagen Party Ltd, Chadstone, Australia) and a 100-well ring, the following qRT-PCR programme was used: 95°C for 30s, 50 cycles of 95°C for 5s and 58°C for 15s. Melting curve analysis was performed to test reaction specificity. Threshold *C*_q_ values and amplification efficiencies were calculated using LinRegPCR (version 2013.0; Ruijter *et al.*, 2009). The qbasePLUS GeNorm software (Integrated Sciences, Chatswood, Australia) validated the reference genes: *Cnot1* and *Dvl1* were suitable for normalization of genes from brain tissue and *Dvl1* and *Ef1α* were selected for gonad gene normalization. Following normalization, the qbasePLUS software was used to produce the final relative quantities of each gene in the brain and gonad and then log2 transformed. Outliers (i.e. individuals with log2 gene expression an order of magnitude different than the mean of the other replicates within a treatment) were removed among all genes assessed in either the brain or gonad.

As *Ef1α* was found suitable for use as a reference gene and because the primers were designed prior to the available *A. polyacanthus* genome or transcriptome, we tested the specificity of the primers. The *Ef1α* target was amplified using 0.2 mM dNTP, 1.5 mM MgCl_2_, 0.2 μM each primer, 1× buffer, 5 units μl^−1^ Taq (Bioline, Alexandria, Australia) and 50 ng cDNA in a C1000 Thermal Cycler (Bio-Rad Laboratories, Gladesville, Australia) with the following steps: pre-denaturation at 94°C for 2 min; 35 cycles of denaturation at 94°C for 45 s, annealing at 61°C for 30 s and extension at 72°C for 20 s; and, finally, a 10 min extension at 72°C. PCR products were visualized on a 1.5% agarose gel and sent to the Australian Genome Research Facility (AGRF) for purification and sequencing. The resulting trimmed 133 bp sequence was compared to those available in the GenBank Public Database (Altschul *et al.*, 1997), with the highest match to Atlantic halibut, *Hippologlossus hippoglossus*, elongation factor 1 alpha, accession EU561358.1, e-value 8e^−17^.

### Statistical analysis

The proportion of mature pairs that reproduced per treatment was compared using a chi squared test of homogeneity among treatments. Generalized least squares (GLS) ANOVA models were used to compare the expression of each gene in the brain and gonad. All samples were first analysed with sex (male or female) and treatment as fixed factors. Following this analysis, separate GLS models were run for male and female gene expression with treatment as a fixed factor. Due to experimental constraints and the nature of the *A. polyacanthus* breeding system, we could not be certain that fish were sampled at the same time within their reproductive cycles. Thus, the time since breeding for each fish was explored as a co-variate in the analysis, but no significant relationship was found. For all analyses, the gls function in the nlme package in R was used (version 3.4.1; Pinheiro *et al.*, 2017).

## Results

### Reproductive success

The proportion of F2 adults that reproduced differed among treatments (*X*^2 ^= 14.06, df = 3, *P* < 0.01). Specifically, 4 of 10 (40%) control pairs, 1 of 9 (11%) developmental pairs, and 6 of 9 (67%) step treatment pairs reproduced, but 0 of 10 (0%) pairs reproduced in the transgenerational treatment. When breeding occurred, there was a tendency for pairs in warm treatments to produce fewer clutches over the season. Specifically, of the four control pairs that reproduced, two produced three clutches over the breeding season and the other two produced one. The single reproductive developmental pair produced two clutches and one of the reproductive step pairs produced two clutches while the other four produced only one.

### Brain gene expression

**Figure 2:**
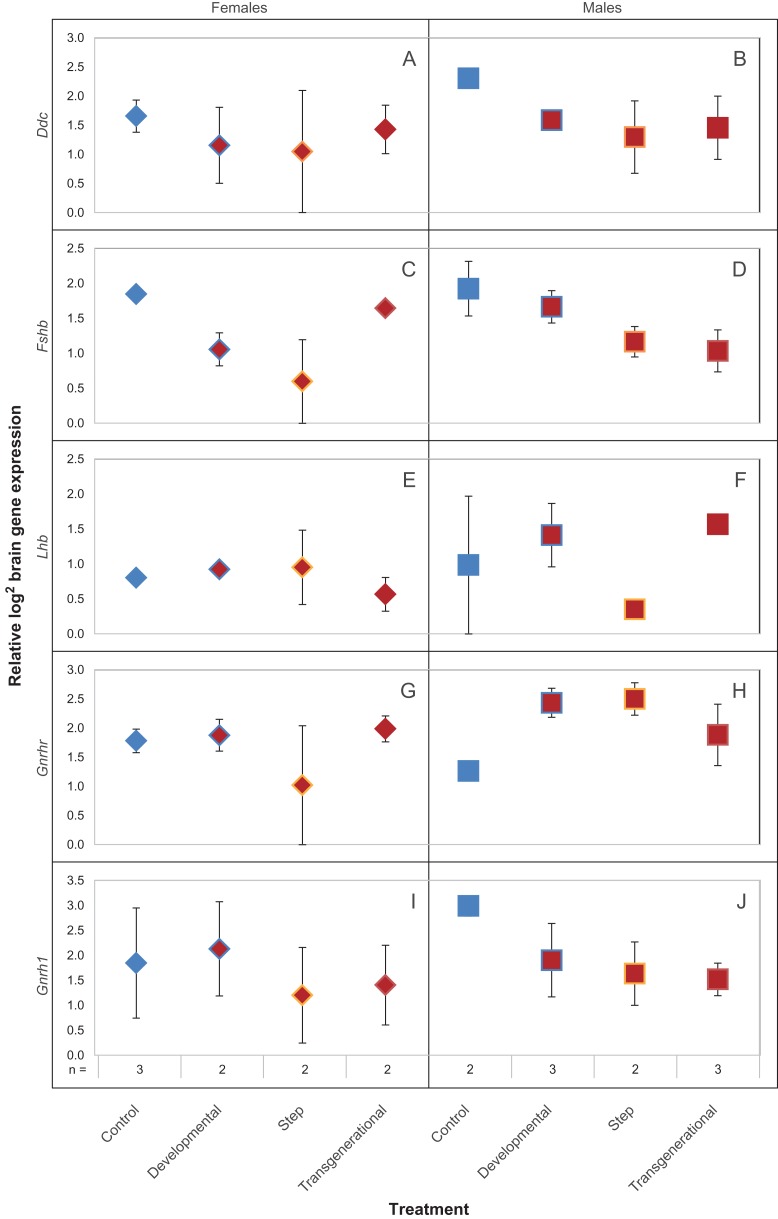
Mean (±SE) log_2_ brain gene expression (*Ddc, Fshb, Lhb, Gnrhr* and *Gnrh1*) for control, developmental, step and transgenerational *Acanthochromis polyacanthus* treatments. Note: some error bars are too small to be seen. Female samples are denoted with diamonds and males with squares. Gene expression relative to reference genes *Cnot1* and *Dvl1*.

The only gene assessed in the brain that exhibited a significant difference in expression depending on treatment was *Fshb*, when all samples were combined (Table 2) and when only females were considered (Table 2; Fig. 2C). Among the females, the significant treatment effect was due to developmental and step treatment fish having 0.8 (±0.3 SE) and 1.3 (±0.6 SE) fold lower expression compared to control, respectively (Fig. 2C). There was no significant effect of treatment in male brain gene expression (Table 2), however *Ddc*, *Fshb* and *Gnrh1* showed decreased trends in expression in temperature treatments relative to control (Fig. 2B, D, J). In contrast, *Gnrhr* had an increased trend in expression in the temperature treatments relative to control, with developmental and step expression both 1.2 (±0.3 SE) fold higher (Fig. 2H).

**Table 2:** Type III analysis of variance (ANOVA) testing differences in brain and gonad gene expression between sexes and/or treatments

Tissue	Gene	ALL				FEMALE				MALE			
		Source	Df	Chisq	Pr(>Chisq)	Source	Df	Chisq	Pr(>Chisq)	Source	Df	Chisq	Pr(>Chisq)
Brain	*Ddc*	(Intercept)	1	14.05	<0.00	(Intercept)	1	10.69	<0.00	(Intercept)	1	24.58	<0.00
		Treatment	3	0.94	0.82	Treatment	3	0.71	0.87	Treatment	3	2.84	0.42
		Sex	1	0.86	0.35								
		Treatment:Sex	3	0.42	0.94								
	*Fshb*	(Intercept)	1	52.77	<0.00	(Intercept)	1	58.63	<0.00	(Intercept)	1	35.20	<0.00
		Treatment	3	11.45	**0.01**	Treatment	3	12.72	**0.01**	Treatment	3	6.02	0.11
		Sex	1	0.04	0.85								
		Treatment:Sex	3	5.82	0.12								
	*Lhb*	(Intercept)	1	5.26	0.02	(Intercept)	1	13.13	<0.00	(Intercept)	1	3.52	0.06
		Treatment	3	0.51	0.92	Treatment	3	1.26	0.74	Treatment	3	3.75	0.29
		Sex	1	0.11	0.74								
		Treatment:Sex	3	3.99	0.26								
	*Gnrh1*	(Intercept)	1	6.64	0.01	(Intercept)	1	4.20	0.04	(Intercept)	1	22.48	<0.00
		Treatment	3	0.71	0.87	Treatment	3	0.45	0.93	Treatment	3	3.68	0.30
		Sex	1	1.01	0.31								
		Treatment:Sex	3	0.80	0.85								
	*Gnrhr*	(Intercept)	1	21.66	<0.00	(Intercept)	1	18.45	<0.00	(Intercept)	1	8.51	<0.00
		Treatment	3	2.65	0.45	Treatment	3	2.26	0.52	Treatment	3	5.76	0.12
		Sex	1	0.73	0.39								
		Treatment:Sex	3	5.61	0.13								
Gonad	*Cyp11b1*	(Intercept)	1	6.92	0.01	(Intercept)	1	8.27	<0.00	(Intercept)	1	124.90	<0.00
		Treatment	3	4.91	0.18	Treatment	3	5.87	0.12	Treatment	3	4.73	0.19
		Sex	1	60.87	**<0.00**								
		Treatment:Sex	3	7.61	0.05								
	*Cyp19a1a*	(Intercept)	1	239.33	<0.00	(Intercept)	1	519.85	<0.00	(Intercept)	1	9.02	<0.00
		Treatment	3	0.46	0.93	Treatment	3	1.01	0.80	Treatment	3	1.65	0.65
		Sex	1	46.22	**<0.00**								
		Treatment:Sex	3	2.30	0.51								
	*Fshr*	(Intercept)	1	15.52	<0.00	(Intercept)	1	9.13	<0.00	(Intercept)	1	167.70	<0.00
		Treatment	3	0.96	0.81	Treatment	3	0.56	0.90	Treatment	3	9.46	**0.02**
		Sex	1	2.28	0.13								
		Treatment:Sex	3	0.48	0.92								
	*Lhcgr*	(Intercept)	1	13.30	<0.00	(Intercept)	1	8.89	<0.00	(Intercept)	1	121.35	<0.00
		Treatment	3	6.96	0.07	Treatment	3	4.65	0.20	Treatment	3	10.94	**0.01**
		Sex	1	9.79	**<0.00**								
		Treatment:Sex	3	10.25	0.795								

Grey fills represent significant results, *P* < 0.05.

### Gonad gene expression

**Figure 3:**
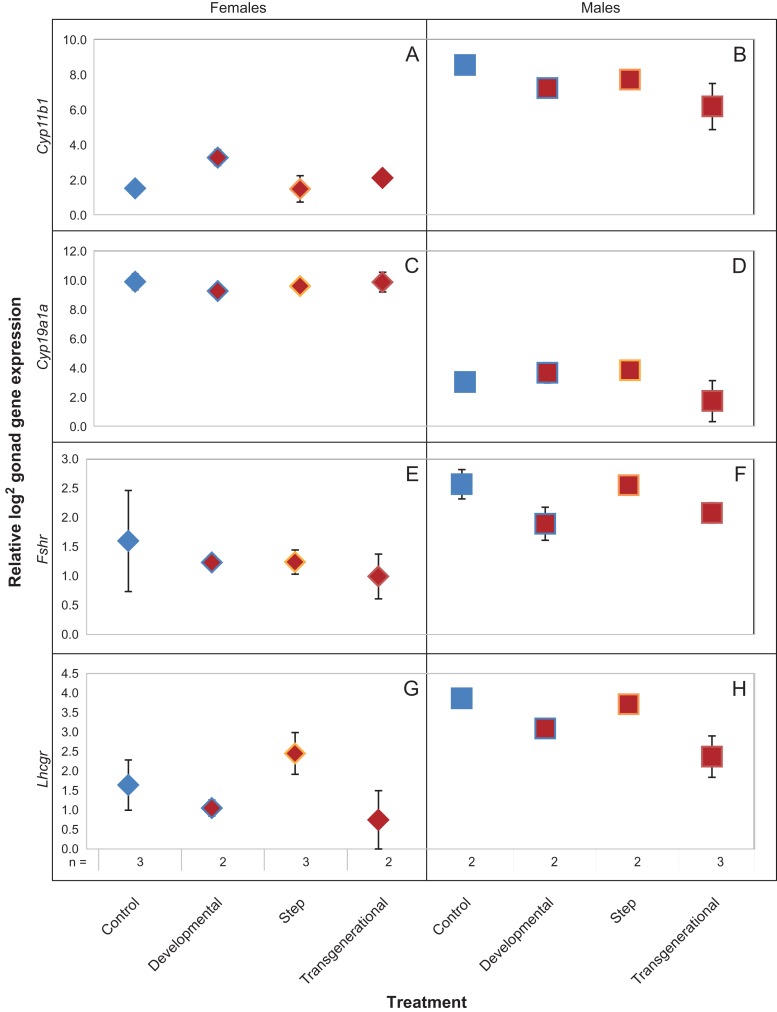
Mean (±SE) log_2_ gonad gene expression (*Cyp11b1*, *Cyp19a1a, Fshr* and *Lhcgr*) for control, developmental, step and transgenerational *Acanthochromis polyacanthus* treatments. Note: some error bars are too small to be seen. Female samples are denoted with diamonds and males with squares. Gene expression relative to reference genes *Dvl1* and *Ef1a*.

*Cyp11b1*, *Cyp19a1a* and *Lhcgr* had significantly different expression between males and females (Table 2): *Cyp19a1a* expression was higher in females (+6.5 fold ± 0.5 SE; Fig. 3C and D) and *Cyp11b1* and *Lhcgr* had elevated expression in males (+5.4 fold ± 0.5 SE and +1.6 fold ± 0.4 SE, respectively; Fig. 3A, B and Fig. 3G, H). There were no significant treatment effects when evaluating all samples (Table 2). There were, however, significant differences among *Fshr* and *Lhcgr* expression in males (Table 2). Male *Fshr* expression in the control and step treatments were higher than developmental and transgenerational treatments (control: +0.7 fold ± 0.4 SE and +0.5 fold ± 0.3 SE, respectively; step: +0.7 fold ± 0.2 SE and +0.5 fold ± 0.1 SE, respectively; Fig. 3F). Similarly, male *Lhcgr* expression in the control and step treatments were higher than developmental and transgenerational treatments (control: +0.8 fold ± 0.3 SE and +1.3 fold ± 0.5 SE, respectively; step: +0.6 fold ± 0.2 SE and +1.2 fold ± 0.4 SE, respectively; Fig. 3H). Expression of both *Fshr* and *Lhcgr* in the males (Fig. 3F and H) showed similar trends to the proportion of pairs that were breeding, with control and step treatments elevated compared to developmental and transgenerational treatments.

There were no significant treatment effects among females (Table 2). Female *Lhcgr* tended to have an elevated trend in expression in step relative to developmental and transgenerational treatments (+1.4-fold ± 0.6 SE and +1.7-fold ± 0.9 SE higher, respectively; Fig. 3G); however, unlike male *Lhcgr*, control fish did not have an increased trend in expression compared to developmental and transgenerational treatments. Female *Cyp11b1* exhibited a trend toward elevated expression in the developmental treatment compared to all other treatments (+1.8-fold ±0.6 SE, +1.8-fold ±0.9 SE, and +1.2-fold ± 0.6 SE vs. control, step, and transgenerational treatments respectively; Fig. 3A).

## Discussion

Maintaining reproductive performance at higher water temperatures will be critical for the persistence of marine species as the climate continues to warm. Although the reef fish *A. polyacanthus* can fully acclimate aerobic metabolism when both parents and offspring are exposed to the same elevated temperatures (Donelson *et al.*, 2012; Veilleux *et al.*, 2015), these fish were unable to reproduce at elevated temperatures; only when there was a more gradual increase in temperature over two generations did fish show improved reproductive capacity (Donelson *et al.*, 2016). Our study aimed to understand how fish were able to adjust reproductive capacity across generations by evaluating the expression of five genes in the brain and four in the gonads of *A. polyacanthus* that are known to be associated with teleost reproduction. By elucidating the molecular mechanisms underpinning reproductive plasticity, we can better understand and predict which populations or species will be most at risk in the future. The step treatment, which experienced a temperature increase of +1.5°C in two successive generations, had a similar proportion of pairs that reproduced compared to control, whereas developmental and transgenerational treatments that were immediately exposed to a +3.0°C increase in temperature had fewer and no pairs reproducing, respectively. Although there were few differences in brain or gonad gene expression among treatments, some patterns emerged. When male and female expression was explored separately, *Fshb* expression in female brains and gonadotropin receptor (*Fshr* and *Lhcgr*) expression in male gonads had significant treatment effects. Although we anticipated that the brain would have a regulatory role in suppressing reproduction in the developmental and transgenerational treatments, of the five genes tested in the brain, only *Fshb* exhibited treatment-specific differences in expression, and the among-treatment pattern did not match expectations for a role in the acclimation of reproduction. Instead, our results suggest that gonadotropin receptors in the male gonads may play a role in the ability to acclimate reproductive capacity and that brain *Fshb* expression could be a temperature-sensitive regulator of vitellogenesis.

Gonadotropins are critical for physiological action and exert their effects on gonads through their receptors, FSH-R and LH-R (Kumar and Trant, 2001). In adult male and female pejerrey, *O. bonariensis*, gonadotropin receptor gene expression decreased when exposed acutely to elevated water temperatures (+4°C and +8°C above the average peak reproductive temperature), though it was only significant for female *Fshr* (Soria *et al.*, 2008). Furthermore, the pejerrey did not spawn at elevated temperatures and had reduced plasma sex steroid levels, leading the authors to suggest that gonads are particularly sensitive to increased water temperatures. In our study, we also observed a decrease in gonadal gonadotropin receptor expression (*Lhcgr* and *Fshr*) in the two elevated temperature treatments that reproduced poorly, developmental and transgenerational, but not the more gradual step treatment, which showed intermediate reproductive capacity. Interestingly, this difference in *Lhcgr* and *Fshr* expression was only found within the male gonads. Donelson *et al.* (2010), found a significant reduction in the proportion of spermatozoa in testes of adult *A. polyacanthus* exposed to +3.0°C for 7 months. As gonadotropins stimulate gamete development and maturation, the elevated levels of receptor expression and superior ability to reproduce at +3.0°C in the step treatment compared to developmental and transgenerational treatments suggests that *Fshr* and *Lhcgr* in the testes may play an important role in plastically altering the ability to reproduce at higher temperatures. Furthermore, *Lhcgr* and *Fshr* in the testes had a similar trend in expression compared to the proportion of pairs that were able reproduce, suggesting that the reduced capacity and inability to reproduce when exposed developmentally or transgenerationally to +3.0°C, respectively, may be due primarily to limitations within the testes and not ovaries. Thus, we have identified testicular *Lhcgr* and *Fshr* as potential biomarkers for reproductive plasticity in *A. polyacanthus*; however, to fully elucidate the role these genes and their encoded proteins play in reproductive capacity and their use as biomarkers, we recommend additional experiments in other species following incremental transgenerational exposure to high temperatures associated with climate change.

Gametogenesis is regulated by the gonadotropins FSH and LH, which are synthesized and released when GnRH stimulates cells of the pituitary gland (Planas and Swanson, 2008; Levavi-Sivan *et al.*, 2010; Zohar *et al.*, 2010). Furthermore, synthesis of the gonadotropins is thought to be dependent on the balance of the activating GnRH and inhibiting DA (Dufour *et al.*, 2010). However, the precise function of FSH and LH in fish gametogenesis is not well understood, exhibiting differences in concentrations at various time points across both synchronous and asynchronous spawners (see Levavi-Sivan *et al.*, 2010 for review). We expected that *Ddc* (a gene encoding an enzyme that converts L-DOPA into DA) and *Gnrh1* would show elevated and reduced expression in the brain, respectively, in the two temperature treatments that had fewer (developmental) and no (transgenerational) pairs capable of reproducing compared to control. However, the trends in brain expression across treatments for *Ddc* and *Gnrh1* were instead similar to each other and also to *Fshb*, showing a general trend of reduced expression in the temperature treatments relative to control. *Fshb* was the only tested gene in the brain in which there was a significant treatment effect and it had reduced expression in treatments exposed to +3.0°C among all individuals and among females. In rainbow trout, FSH stimulated the incorporation of over twice as much vitellogenin into ovaries compared to LH (Tyler *et al.*, 1991) and plays a primary role in mediating vitellogenic development (Tyler *et al.*, 1997). In yellowtail kingfish treated with recombinant FSH, females exhibited an increased trend in oocyte diameter compared to controls (Sanchís-Benlloch *et al.*, 2017). Donelson *et al.* (2016) found that when a subset of the transgenerational *A. polyacanthus* were transferred to control temperatures during reproduction, they produced significantly smaller eggs compared to control. Thus, the reduction in *Fshb* in female *A. polyacanthus* +3.0°C treatments suggests that FSH, a regulator of vitellogenesis, is sensitive to increases in temperature, regardless of whether experienced developmentally, step-wise or transgenerationally.

The observed expression profile differences of *Cyp11b1* and *Cyp19a1a* between sexes is intuitive: *Cyp11b1* is 5.4-fold significantly greater in males as its encoded protein converts testosterone to the active metabolite 11-KT, while *Cyp19a1a* is 6.5-fold greater in females as its encoded protein converts testosterone to E_2_. There was no difference in the expression of *Cyp19a1a* among treatments in females, despite reduced *Fshb* expression in the higher temperature groups. Similarly, coho salmon ovarian follicles incubated with FSH showed no difference in *Cyp19a1a* compared to control, but had significantly elevated E_2_ levels (Luckenbach *et al.*, 2011). The authors suggested that increases in E_2_, but not *Cyp19a1a*, could be due to upregulation of other genes in the steroid biosynthesis cascade. In thermally stressed Atlantic salmon, *Salmo salar*, there were reductions in egg size, which was associated with reductions in plasma E_2_ levels (King *et al.*, 2003). Thus, despite no change in *Cyp19a1a* among *A. polyacanthus* females, FSH may still cause a reduction in E_2_ levels in the elevated temperature treatments by affecting expression of other genes in the steroid biosynthesis cascade, ultimately leading to reduced egg size.

Consistent with the results from Donelson *et al.* (2016), here we show that *A. polyacanthus* can acclimate reproductive capacity to +3.0°C if temperature is ramped in +1.5°C steps across generations. This pattern of improved reproduction matched the observation that male gonadotropin receptor (*Lhcgr* and *Fshr*) gene expression in the gonads in the step treatment were at control levels, and higher than the two other elevated temperature treatments. This difference in *Lhcgr* and *Fshr* gene expression in male rather than female gonads suggests that spermatogenesis may be more thermosensitive than oogenesis in *A. polyacanthus*. Furthermore, the expression pattern of *Lhcgr* and *Fshr* indicate that plasticity of these genes within the testes may improve reproductive capacity and further research into the molecular mechanisms leading to improved spermatogenesis following gradual transgenerational exposure to increased temperatures should be explored. The fish used in this study were sacrificed on the same date and thus not all individuals were at the same reproductive stage. Therefore, although *Fshb*, *Fshr* and *Lhcgr* showed significant differences among treatments, the other genes assessed that were not significant (*Ddc*, *Lhb*, *Gnrh1*, *Gnrhr*, *Cyp11b1* and *Cyp19a1a*) may still play a role in plasticity, but at more specific time points during the reproductive cycle. Future studies could examine the expression of these genes throughout the reproductive cycle to test for a possible role in reproductive acclimation to elevated temperatures. Here we identified the sex, tissue, and genes that are likely involved in transgenerational plasticity of reproductive capacity in *A. polyacanthus*, thus providing a more targeted approach for assessing the effects of increased temperature on this species and others, in both wild and laboratory settings. Furthermore, our findings highlight the need for experimental approaches that increase temperature gradually, or in several steps, to better understand how species will cope with future climate change over relevant time scales.
